# Long-term results of radiotherapy for periarthritis of the shoulder: a retrospective evaluation

**DOI:** 10.1186/1748-717X-2-34

**Published:** 2007-09-14

**Authors:** Marcus Niewald, Jochen Fleckenstein, Susanne Naumann, Christian Ruebe

**Affiliations:** 1 Dept. of Radiooncology, Saarland University Hospital, Kirrberger Str.1, D-66421, Homburg, Germany

## Abstract

**Background:**

To evaluate retrospectively the results of radiotherapy for periarthritis of the shoulder

**Methods:**

In 1983–2004, 141 patients were treated, all had attended at least one follow-up examination. 19% had had pain for several weeks, 66% for months and 14% for years. Shoulder motility was impaired in 137/140 patients. Nearly all patients had taken oral analgesics, 81% had undergone physiotherapy, five patients had been operated on, and six had been irradiated. Radiotherapy was applied using regular anterior-posterior opposing portals and Co-60 gamma rays or 4 MV photons. 89% of the patients received a total dose of 6 Gy (dose/fraction of 1 Gy twice weekly, the others had total doses ranging from 4 to 8 Gy. The patients and the referring doctors were given written questionnaires in order to obtain long-term results. The mean duration of follow-up was 6.9 years [0–20 years].

**Results:**

During the first follow-up examination at the end of radiotherapy 56% of the patients reported pain relief and improvement of motility. After in median 4.5 months the values were 69 and 89%, after 3.9 years 73% and 73%, respectively. There were virtually no side effects. In the questionnaires, 69% of the patients reported pain relief directly after radiotherapy, 31% up to 12 weeks after radiotherapy. 56% of the patients stated that pain relief had lasted for "years", in further 12% at least for "months".

**Conclusion:**

Low-dose radiotherapy for periarthropathy of the shoulder was highly effective and yielded long-lasting improvement of pain and motility without side effects.

## Background

The application of roentgen rays to the joints has been known since the end of the 19^th ^century and was found to be successful even more than 70 years ago [1,2]. In the following decades, radiotherapy for benign diseases was widely accepted in Germany, Switzerland and Austria, while these techniques were rarely utilized in other West European countries for fear of an elevated frequency of secondary malignancies [3,4]. In general inflammatory or degenerative disorders of the joints or the surrounding tendons are treated with very low total doses of ionizing radiation in order to achieve pain relief and improvement of the joint motility [1,5,6]. Periarthritis of the shoulder is a rather frequent disease belonging to this group. In the last ten years the general periarthritis humeroscapularis (PHS) has been subdivided into several syndromes. According to the classification published by Hedtmann et al. [7] a simple, an adhesive, a calcifying, and a destructive PHS should be distinguished. In our series all patients had been diagnosed with a calcifying PHS (calcific tendinosis or tendinitis).

Etiology and pathogenesis of this disease are still not understood completely. Mechanical, traumatic, metabolic, circulatoric, thermic, infectious, toxic and psychical factors may lead to degenerative changes of the tendons and ligaments, with secondary calcifications. These may initiate local inflammative processes causing pain and impairment of mobility [7-10].

For treatment, oral analgesics are applied as well as injection of corticosteroids into the affected region. Physiotherapy is recommended generally, often consisting of special gymnastic exercises, electrotherapy or the application of cold or hot packs. Eventually, surgical interventions may become necessary.

The purpose of this study was to examine whether radiotherapy is effective in the treatment of shoulder periarthropathy and thus can be a reasonable alternative to the other therapeutic methods mentioned above.

## Methods

In the time interval 1983–2004, a total of 141 patients were irradiated for periarthritis of the shoulder, especially calcifying PHS as defined in the Kraemer/Hedtmann [7] classification. The diagnosis was based on anamnesis, orthopaedic examination with typical findings and a conventional X-ray examination showing calcifications within the tendon of the supraspinatus muscle.

Among the patients were 70 men and 71 women, the mean age at the beginning of therapy was 57 years [27–90 years]. All patients suffered from pain, 27(19%) had been for some weeks, 93(66%) for some months, 20(14%) for some years (no data for one patient). In 137/140 (98%) patients an impairment of shoulder mobility was known, in 7/141 (5%) a local swelling, in 8/135 (6%) an intraarticular effusion, and in 14/139 (10%) patients a traumatic lesion was known (the figures show the number of patients showing a special finding in comparison to the number where information is available). 107/132 (81%) patients had undergone physiotherapy, while a puncture of the shoulder joint had been performed in 8/135 (6%) patients, 5/128 (4%) had been operated on, 6/138 (3%) had been irradiated. Nearly all patients had received oral medication with non-steroidal analgesics, Corticosteroid injections had been performed in 66/129 (51%) patients.

**Figure 1:**
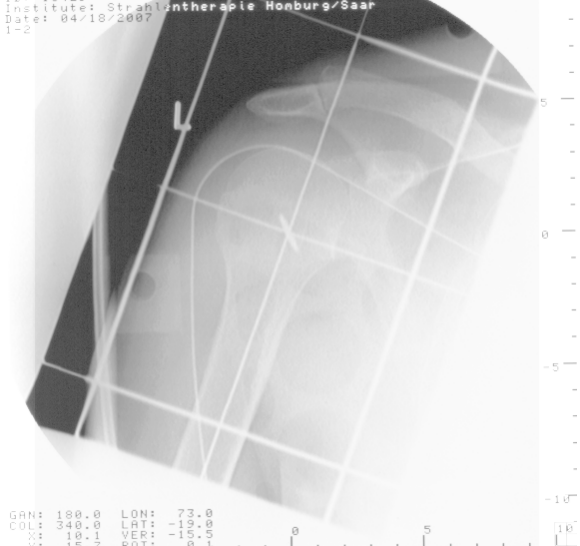
**Typical radiotherapy field**. A/p simulator radiograph of a typical radiotherapy portal (with kind permission by the patient)

In 137/141 (97%), treatment was performed using regular and mainly isocentric ap/pa opposing fields with a mean field length of 13 cm [5.5–20 cm] and a mean field width of 13 cm [6–22.5 cm] (see Fig. 1) in supine position (up to 1987, the anterior field was treated in supine position, the posterior one in prone position). The remaining four patients (3%) received anterior fields of comparable size. The beam qualities, total doses and doses per fraction used have been summarized in Table 1. All patients were irradiated twice a week.

**Table 1:** Patient collective

Item	Number of patients	Percentage
*Beam qualitites*		
Co-60	52	
4 MV photons	52	
6 MV photons	33	
Electrons	2	
Orthovoltage	2	
Total	141	100
		
*Total dose [Gy]*		
4.0	1	0.7
5.0	2	1.4
6.0	123	87.2
6.5	1	0.7
7.0	2	1.4
8.0	12	8.6
Total	141	100
		
*Dose/fraction [Gy]*		
0.5	5	3.6
1	133	94.3
2	1	0.7
7	2	1.4
Total	141	100

Summary of the beam qualities used, of the total doses and the doses per fraction.

The first follow-up examination was scheduled at the last day of radiotherapy, further examinations 6 weeks afterwards, and after that every three to six months.

The patients' records were evaluated meticulously. A vast majority of them did not attend regular follow-up examinations, so that written questionnaires were mailed to the patients and the referring doctors in order to achieve additional data concerning frequency and duration of pain relief or improvement of mobility as well as to see if any side effects had been noticed.

Improvement of pain was graded according to the classification published by von Pannewitz [1] in 1933 (painless, markedly improved, improved, stable, worse).

All data were entered into a special medical database (MEDLOG, Parox Comp., Muenster, Germany). Absolute and relative frequencies were computed. The search for prognostic factors was performed univariately using Spearman's rho and Kendall's tau tests as well as multivariately using the Cox regression hazard model.

All patients had given their written informed consent before radiotherapy. An approval by the local ethics committee was not necessary due to the retrospective evaluation. The research having been carried out here is in compliance with the declaration of Helsinki.

## Results

At least one set of reliable follow-up information was available from all 141 patients. During evaluation it was noted that the patients either did not attend the scheduled follow-up examination at all or not within the time intervals scheduled.

**Figure 2:**
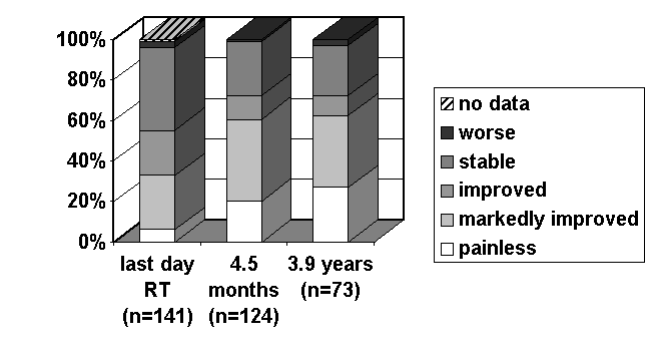
**Pain relief versus time**. Percentage of patients with a certain result concerning pain relief according to the von Pannewitz classification at the last day of radiotherapy, in median 4.5 months later and in median 3.9 years later

**Figure 3:**
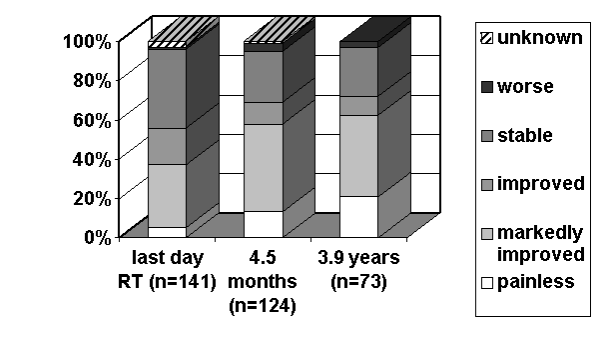
**Improvement of mobility versus time**. Percentage of patients with a certain result concerning improvement of mobility according to the von Pannewitz classification at the last day of radiotherapy, in median 4.5 months later and in median 3.9 years later

As stated earlier, one follow-up dataset (including the results of the questionnaires) was available from 141 patients, two from 124 patients and three from 73 patients. The first follow-up examination took place in median at the end of radiotherapy, the second one after in median 139 days (4 1/2 months) while the third set of information was obtained in median 3.9 years after therapy. The detailed data concerning pain relief and improvement of mobility are given in figures 2 and 3. In summary, directly after radiotherapy 56% of the patients experienced pain relief, the same percentage noticed an improvement of joint mobility. The figures for the time points of 4.5 months and of 3.9 years after radiotherapy amount to 69% and 73%, respectively. Among the seven patients with joint swellings, three noticed an improvement directly after radiotherapy, and five in median 4.5 months afterwards.

135 patients returned their questionnaires, alternatively they were interviewed during a follow-up examination or by phone. Their answers concerning the time of onset of improvement, duration of improvement and overall satisfaction are summarized in Table 2.

**Table 2:** Patients' opinions in the questionnaires

**Item**	**Absolute frequency**	**Relative frequency (%)**
Time of onset of improvement (n = 109)		
During RT	1	1
End of RT	65	60
> 2 weeks after	5	5
> 4 weeks after	1	1
> 8 weeks after	10	9
>12 weeks after	27	24
		
Duration of improvement (n = 135)		
Not at all	29	21
For weeks	14	10
For months	16	12
For years	76	57
		
Overall patients' satisfaction (n = 86)		
satisfied	51	59
unsatisfied	27	31
no opinion	8	10

Summary of the patient's data concerning onset and duration of improvement and overall satisfaction

The only side effect was a mild redness of skin after radiotherapy (acute dermatitis 1° according to the classification of the World Health Organization) in one patient.

After radiotherapy, 53/121 patients had no further treatment. In a further 52 physiotherapy was continued, five were operated on and the remaining 11 underwent a second series of radiotherapy.

We did not succeed in finding independent prognostic factors for pain relief either univariately or multivariately.

## Discussion

Bearing in mind the well known limits of a retrospective evaluation and the partially incomplete database, we think that radiotherapy for periarthritis of the shoulder has been shown to be an effective method in order to achieve pain relief and improvement of mobility of the shoulder joint in our patient series. Our results fit well to those published in the literature (see Table 3) [11-28]. Side effects were never reported there. Unfortunately, it was not possible to perform an detailed statistical analysis of absolute pain scores before vs. after radiotherapy. As stated earlier, our patients have been treated in the years 1983 to 2004, in the earlier years of this time interval no pain scores have been used, the patients have only been re-examined for improvement. Nevertheless, these relative data are regarded reliable, as the improvement data at different time points in follow up are correlated highly significantly with each other (p < 0.001, Spearman's Rho and Kendall's Tau).

**Table 3:** Literature data

Authors	Number of patients	Parameter	Dose per fraction/total dose	Time of data collection	Results (%, Pannewitz class.)			
					
					+++	++	+	0
Fuchs u. Hofbauer (1957)^11^	28	Pain Mobility	60 – 100 R/600 – 1500 R	End of RT	79%	17%	--	4%
Braun u. Jakob (1965)^12^	25	Pain Mobility	100 – 140 R/300 – 1640 R	Not stated	64%	32%	--	4%
Schertel (1968)^13^	89	Pain Mobility	100 R/400 – 600 R	6 weeks after RT	2%	18%	43%	49%
Wieser (1969)^14^	160	Pain	40 – 120 R/500 R	End of RT	22%	45%	22%	11%
Keinert (1972)^15^	145	Pain Mobility	30 – 100 R/400 R	Several weeks after RT	50%	41%	--	9%
Zilberberg et al. (1976)^16^	200	Pain Mobility	120 R/1200 R	4 weeks after RT	46%	24%	16%	14%
Hassenstein (1979)^17^	233	Pain Mobility	0.5 – 1 Gy/1.5 – 3 Gy	4–6 weeks after RT	43%	31%	--	26%
Goerlitz (1981)^18^	50	Pain Mobility	0.5 Gy/4 Gy	3 months after RT	48%	34%	--	18%
Hess (1988)^19^	164	Pain	0.3 – 0.5 Gy/up to 3 Gy	Several time intervals	49%	27%	--	24%
Sautter-Bihl (1993)^20^	30	Pain Mobility	0.5 – 1 Gy/2.5 – 6 Gy	End of RT	33%	27%	27%	13%
Keilholz et al. (1995)^21^	106	Orthopedic scores	0.5 Gy/3 Gy	6 weeks after RT	49%	32%	--	19%
Schaefer u. Micke (1996)^22^	42	Pain Mobility	0.5 – 1 Gy/2 – 4 Gy	6 weeks after RT	61%	15%	--	24%
Heyd (1998)^23^	41	Pain Mobility	1 Gy/4 Gy	Several time intervals	44%	27%	17%	12%
Seegenschmiedt (1998)^24^	89	Pain Orthop. Scores	0.5/6 Gy (2 × 3 Gy)	6 weeks after RT	49%	26%	6%	19%
Zwicker et al. (1998)^25^	77	Pain Mobility	1 Gy/6 Gy	3 months after RT	34%	35%	20%	11%
Schultze (2004)^26^	94	Pain Mobility	0.75 Gy/6 Gy	4 months after RT	18%	27%	14%	41%
Own results	141	Pain Mobility	1.0 Gy/6 Gy	4.5 months after RT	19% 13%	39% 45%	11% 11%	31% 30%

Comparison of literature data with our results

Explanation of abbreviations:

+++: painless

++: markedly improved

+: improved

0: stable or worse

Some author groups regard a follow-up duration of at least 6 months [19-21,24] very important in order to achieve reliable results. This challenge could easily be met in our data. A further question in the literature was whether a longer-lasting pain anamnesis is correlated with a worse prognosis. In our data, duration of previous pain could not be identified as a prognostic factor, the findings in the literature are contradictory [17,19,21].

The underlying mechanism of radiotherapy with small doses is not yet understood completely. More than 50 years ago, Hornykiewytsch et al. [29] found that exposing tissue to Roentgen rays first led to a tissue acidosis and later to a longer-lasting alkalosis, this finding was regarded to be one of the mechanisms for pain relief for a long time. More recent experiments showed that artificial arthritis in rodents and canines responded well to low doses of radiation, based on a reduced expression of inflammatory cytokines and an increased apoptosis of monocytes without secretion of inflammatory cytokines from those cells. Furthermore, according to Trott et al. [30], radiation may have effects on the inducible nitric oxide synthase activity. An anti-proliferative effect was noted solely after radiotherapy with higher doses of 10 Gy and above [1,27,28,30-36].

We have found only one author group which has compared different doses of radiotherapy in a randomized trial (Hassenstein et al., 1979[17]). They found significantly better results in patients applied greater doses than 1.5 Gy.

Alternative treatment methods have been discussed. One of the oldest of these is the local injection of corticosteroids. Keilholz et al. [21] reported a success rate of as high as 90%, but there to a risk of local complications such as infections, necrosis and tendon ruptures especially after multiple injections.

Surgery consisting of a widening of the subacromial space, suturing of the injured tendon or removal of the calcified plaques can lead to a rate of pain relief up to 85%. However, physiotherapy is recommended for 8–12 weeks after the operation [8,9]. Rupp et al. [37] reviewed the modern surgical possibilities in more detail. Needling of the shoulder joint, arthroscopic and open surgery have been found to yield comparable results concerning the resorption of the calcified plaques and – only secondary – pain relief. Unfortunately, the modern radiotherapeutic literature was not taken into account by the authors, so that the conclusion that radiotherapy cannot be recommended in general is debatable. Seil et al. [38] concluded in their retrospective evaluation that arthroscopic surgery is successful in more than 90% of the patients, pain relief was slowly progressive and sometimes even for a period of one year.

Besides a lot of retrospective data concerning extracorporeal shock wave therapy (ESWT), there are two randomized trials which have shown the superiority of high-energy ESWT (Loew et al. 1999 [39], Consentino et al. 2003 [40]) compared to analgesics. Rupp and Seil [41,42] reviewed the effects of ESWT in general and compared different treatment schedules in detail. After a follow-up of 6 months, they found a plaque resorption rate ranging from 34–48% depending on the number and energy of shock wave impulses. The group of patients with resorption of calcified plaques after therapy showed significantly better results concerning improvement of mobility and pain relief compared to the group without resorption.

The effect of local laser treatment has been tested recently by Bingol et al [43] in a randomized trial. They found no increased effect on pain and active mobility of laser application combined with a special exercise program compared to placebo laser treatment and the same exercises, whereas the sensitivity to palpation and the passive mobility were improved.

To our knowledge, radiotherapy has only once been compared to any alternative method in a randomized trial in the literature (Haake et al., 2001 [44], Gross et al., 2002 [45]). They compared a radiation dose of 3 Gy in fractions of 0.5 Gy with extracorporal shock wave therapy in 30 patients and found equal efficacy of both methods.

## Conclusion

In our series, low-dose radiotherapy for painful periarthritis of the shoulder was found to be an important therapeutic alternative to medication, injections, ESWT and surgery because of a high rate of long-lasting pain relief and improvement of mobility with virtually no side effects. A recent investigation of the efficacy of radiotherapy in a randomized trial is still lacking as is the comparison with alternative methods in large trials. The next step initiated by the German Cooperative Group on Benign Diseases (GCGBD) of the DEGRO (German Society on Radiation Oncology) will be a Patterns-of-care-study [3] in order to get an overview of the therapeutic possibilities, methods and results all over Germany. After that, a randomized trial is urgently required comparing radiotherapy with best supportive care or with another therapy method.

## Competing interests

The author(s) declare that they have no competing interests.

## Authors' contributions

MN was responsible for the conception and design of the study, check of the data, statistical evaluation, and writing of the manuscript. JF was responsible for the treatment of the majority of the patients, control of documentation of treatment and follow-up data, and review of the manuscript. SN was responsible for the evaluation of the patients' records, collection of the data, letters to the patients and the referring doctors, and the entry of the data to the databank system. CR critically evaluated and approved the manuscript.

All authors have read and approved the final manuscript.

## References

[B1] Von Pannewitz G, Holfelder H, Holthausen H, Juengling O, Martius H, Schinz HR (1933). Roentgen therapy for deforming arthritis. Ergebnisse der medizinischen Strahlenforschung.

[B2] Reichel WS (1949). Die Roentgentherapie des Schmerzes. Strahlenther Onkol.

[B3] Seegenschmiedt MH, Katalinic A, Makoski H, Haase W, Gademann G, Hassenstein E (2000). Radiation therapy for benign diseases: Patterns of care study in Germany. Int J Radiat Oncol Biol Phys.

[B4] Leer JW, van Houtte P, Davelaar J (1998). Indications and treatment schedules for irradiation of benign diseases: a survey. Radiother Oncol.

[B5] Von Pannewitz G, Zuppinger A, Ruckensteiner E (1970). Degenerative Erkrankungen. Handbuch der Medizinischen Radiologie.

[B6] Ernst-Stecken A, Sauer R, Seegenschmiedt MH, Makoski HB (1988). Degenerative Erkrankungen: Insertionstendopathien. Radioonkologisches Kolloquium: Radiotherapie von gutartigen Erkrankungen.

[B7] Hedtmann A, Fett H (1989). So-called humero-scapular periarthropathy – classification and analysis based on 1,266 cases. Z Orthop Ihre Grenzgeb.

[B8] Eulert J (1977). Periarthritis. Pathogenesis, clinical picture and treatment of the so-called periarthritis humero-scapularis. ZFA (Stuttgart).

[B9] Eulert J, Apoil A, Dautry P (1981). Pathogenesis and surgical treatment of "periarthritis humeroscapularis" (author's transl). Z Orthop Ihre Grenzgeb.

[B10] Jerosch J (1996). Periarthritis humeroscapularis – clinical diagnosis and analysis of the syndrome concept. Wien Med Wochenschr.

[B11] Fuchs G, Hofbauer J (1957). [Roentgen therapy of periarthritis humero-scapularis.]. Wien Klin Wochenschr.

[B12] Braun H, Jacob KO (1965). [Roentgen therapy of periarthritis humeroscapularis]. Med Klin.

[B13] Schertel L, Roos A (1968). [Radiotherapy in degenerative skeletal diseases]. Med Klin.

[B14] Wieser C (1969). [Roentgen irradiation of the painful shoulder]. Praxis.

[B15] Keinert K, Schumann E, Grasshoff S (1972). [Radiotherapy of humeroscapular periarthritis]. Radiobiol Radiother (Berl).

[B16] Zilberberg C, Leveille-Nizerolle M (1976). [Anti-inflammatory radiotherapy in 200 cases of scapulo-humera 1 peri-arthritis]. Sem Hop.

[B17] Hassenstein E, Nusslin F, Hartweg H, Renner K (1979). [Radiation therapy of humeroscapular periarthritis (author's transl)]. Strahlenther.

[B18] Goerlitz N, Schalldach U, Roessner B (1981). Die Strahlentherapie der Periarthropathia humeroscapularis and Epicondylitis humeri. Dtsch Gesundheitswesen.

[B19] Hess F, Schnepper E (1988). [Success and long-term results of radiotherapy of periarthritis humeroscapularis]. Radiologe.

[B20] Sautter-Bihl ML, Liebermeister E, Scheurig H, Heinze HG (1993). [Analgetic irradiation of degenerative-inflammatory skeletal diseases. Benefits and risks]. Dtsch Med Wochenschr.

[B21] Keilholz L, Seegenschmiedt MH, Kutzki D, Sauer R (1995). [Periarthritis humeroscapularis (PHS). Indications, technique and outcome of radiotherapy]. Strahlenther Onkol.

[B22] Schaefer U, Micke O, Willich N (1996). Schmerzbestrahlung bei degenerativ bedingten Skeletterkrankungen. Roentgenpraxis.

[B23] Heyd R, Schopohl B, Bottcher HD (1998). [Radiation therapy in humero-scapular peri-arthropathy. Indication, method, results obtained by authors, review of the literature]. Roentgenpraxis.

[B24] Seegenschmiedt MH, Keilholz L (1998). Epicondylopathia humeri (EPH) and peritendinitis humeroscapularis (PHS): evaluation of radiation therapy long-term results and literature review. Radiother Oncol.

[B25] Zwicker C, Hering M, Brecht J, Bjornsgard M, Kuhne-Velte HJ, Kern A (1998). [Radiotherapy of humero-scapular periarthritis using ultra-hard photons. Evaluation by MRI findings]. Radiologe.

[B26] Schultze J, Schlichting G, Galalae R, Koltze H, Kimmig B (2004). [Results of radiation therapy in periarthritis humeroscapularis]. Roentgenpraxis.

[B27] Hildebrandt G, Seed MP, Freemantle CN, Alam CA, Colville-Nash PR, Trott KR (1998). Mechanisms of the anti-inflammatory activity of low-dose radiation therapy. Int J Radiat Oncol Biol Phys.

[B28] Hildebrandt G, Maggiorella L, Roedel F, Roedel V, Willis D, Trott KR (2002). Mononuclear cell adhesion and cell adhesion molecule liberation after X-irradiation of activated endothelial cells in vitro. Int J Radiat Oncol Biol Phys.

[B29] Hornikiewytsch T (1952). Physikalisch-chemische und histochemische Untersuchungen ueber die Wirkung von Roentgenstrahlen. Strahlenther Onkol.

[B30] Trott KR, Kamprad F (1999). Radiobiological mechanisms of anti-inflammatory radiotherapy. Radiother Oncol.

[B31] Kern B, Keilholz L, Forster C, Seegenschmiedt MH, Sauer R, Herrmann M (1999). In vivi apoptosis in peripheral blood mononuclear cells induced by low-dose radiotherapy displays a discontinuous dose-dependance. Int J Radiat Oncol Biol Phys.

[B32] Roedel F, Kamprad F, Sauer R, Hildebrandt G (2002). Funktionelle und molekulare Aspekte der antiinflammatorischen Wirkung niedrig dosierter Radiotherapie. Strahlenther Onkol.

[B33] Roedel F, Kley N, Beuscher HU, Hildebrandt G, Keilholz L, Kern P, Voll R, Herrmann M, Sauer R (2002). Anti-inflammatory effect of lose-dose X-irradiation and the involvement of a TGFβ1-induced down-regulation of leucocyte/endothelial cell adhesion. Int J Radiat Oncol Biol Phys.

[B34] Fischer U, Kamprad F, Koch F, Ludewig E, Melzer R, Hildebrandt G (1998). Effekte einer niedrig dosierten Co-60-Bestrahlung auf den Verlauf einer aseptischen Arthritis am Kniegelenk des Kaninchens. Strahlenther Onkol.

[B35] Steffen C, Mueller Ch, Stellamor K, Zeitlhofer J (1982). Influence of X-ray treatment on antigen-induced experimental arthritis. Ann Rheum Dis.

[B36] Trott KR, Parker R, Seed MP (1995). Die Wirkung von Roentgenstrahlen auf die experimentelle Arthritis der Ratte. Strahlenther Onkol.

[B37] Rupp S, Seil R, Kohn D (2000). [Tendinosis calcarea of the rotator cuff]. Orthopaede.

[B38] Seil R, Litzenburger H, Kohn D, Rupp S (2006). Arthroscopic treatment of chronically painful calcifying tendinitis of the supraspinatus tendon. Arthroscopy.

[B39] Loew M, Daecke W, Kusnierczak D, Rahmanzadeh M, Ewerbeck V (1999). Shock-wave therapy is effective for chronic calcifying tendinitis of the shoulder. J Bone Joint Surg Am.

[B40] Consentino R, DeStefano R, Selvi E, Frati E, Manca S, Frediani B, Marcolongo R (2003). Extracorporal shock wave therapy for chronic calcific tendinitis of the shoulder: single blind study. Ann Rheum Dis.

[B41] Seil R, Rupp S, Hammer DS, Ensslin S, Gebhardt T, Kohn D (1999). [Extracorporeal shockwave therapy in tendionosis calcarea of the rotator cuff: comparison of different treatment protocols]. Z Orthop Ihre Grenzgeb.

[B42] Rupp S, Gebhardt T, Kohn D (1998). Die extrakorporale Stoßwellentherapie (ESWT) am Bewegungsapparat. Saarlaendisches Aerzteblatt.

[B43] Bingol U, Altan L, Yurtkuran M (2005). Low-power laser treatment for shoulder pain. Photomed Laser Surg.

[B44] Haake M, Sattler A, Gross MW, Schmitt J, Hildebrandt R, Muller HH (2001). [Comparison of extracorporeal shockwave therapy (ESWT) with roentgen irradiation in supraspinatus tendon syndrome – a prospective randomized single-blind parallel group comparison]. Z Orthop Ihre Grenzgeb.

[B45] Gross MW, Sattler A, Haake M, Schmitt J, Hildebrandt R, Muller HH, Engenhart-Cabillic R (2002). [The effectiveness of radiation treatment in comparison with extracorporeal shockwave therapy (ESWT) in supraspinatus tendon syndrome]. Strahlenther Onkol.

